# Repetitive transcranial magnetic stimulation induces long-lasting changes in protein expression and histone acetylation

**DOI:** 10.1038/srep16873

**Published:** 2015-11-20

**Authors:** Adeline Etiévant, Stella Manta, Camille Latapy, Luiz Alexandre V. Magno, Shirley Fecteau, Jean-Martin Beaulieu

**Affiliations:** 1 Department of psychiatry and neuroscience, Faculty of medicine, Université Laval. Québec City, Qc, Canada; 2 Universidade Federal de Minas Gerai, Belo Horizonte, Brazil; 3 Departement of readaptation, Faculty of medicine, Université Laval, Québec City, Qc, Canada and Berenson-Allen Center for Noninvasive Brain Stimulation, Beth Israel Deaconess Medical Center, Harvard Medical School, Boston, Massachusetts, United States of America

## Abstract

The use of non-invasive brain stimulation like repetitive transcranial magnetic stimulation (rTMS) is an increasingly popular set of methods with promising results for the treatment of neurological and psychiatric disorders. Despite great enthusiasm, the impact of non-invasive brain stimulation on its neuronal substrates remains largely unknown. Here we show that rTMS applied over the frontal cortex of awaken mice induces dopamine D_2_ receptor dependent persistent changes of CDK5 and PSD-95 protein levels specifically within the stimulated brain area. Importantly, these modifications were associated with changes of histone acetylation at the promoter of these genes and prevented by administration of the histone deacetylase inhibitor MS-275. These findings show that, like several other psychoactive treatments, repeated rTMS sessions can exert long-lasting effects on neuronal substrates. This underscores the need of understanding these effects in the development of future clinical applications as well as in the establishment of improved guidelines to use rTMS in non-medical settings.

High-frequency repetitive transcranial magnetic stimulation (rTMS) over the left dorsolateral prefrontal cortex (DLPFC) is an FDA approved therapeutic intervention for drug-resistant major depression^1^,^2^. rTMS has also shown promising results for several other neuropsychiatric and neurological disorders such as substance use disorders, schizophrenia and post-traumatic stress disorder^2^,^3^,^4^,^5^,^6^,^7^. Furthermore, due to their apparent benign nature rTMS and other forms of noninvasive brain stimulation are seriously considered means to modulate cognitive functions such as decision-making processes in healthy individuals^8^,^9^. Despite its growing popularity, the cellular and molecular mechanisms underlying rTMS long lasting effects on brain functions remain for the most part unknown. A better understanding of the molecular actions of rTMS is thus needed to improve its clinical benefits, guide its rational combination with existing and experimental drug therapies and establish secure guidelines for its use in different human populations.

It has been postulated that some of the effects of rTMS on brain circuitry may be associated with long-term potentiation/depression (LTP/LTD)-like mechanisms^10^,^11^. Furthermore, rTMS has been shown to increase both brain derived neurotrophic factor (BDNF)^11^,^12^,^13^ and dopamine^14^,^15^,^16^,^17^ release transiently after stimulation. In line with this, some studies reported a possible association between the Val 66 Met BDNF gene variant and rTMS treatment efficacy^18^,^19^. That being said, these effects are transient and little is known about the potential long-lasting impact of rTMS treatment on neuronal functions.

Chronic treatment with several psychoactive drugs, including selective serotonin reuptake inhibitors (SSRI) and psychostimulant drugs such as cocaine, which act on dopamine neurotransmission, have been shown to exert part of their actions by inducing epigenetic modulation of chromatin organization leading to changes in gene expression^20^,^21^,^22^. Such effects are long-lasting and can affect neuronal functions and behavior from weeks to years after drug withdrawal. One mechanism of epigenetic modulation involves the modulation of histone H3 and H4 acetylation by two families of enzymes that are tightly co-regulated at equilibrium^23^. As indicated by their name, histone acetyl-transferases (HAT) mediate the acetylation of histones. This results in an uncoiling of the chromatin that renders DNA more accessible for transcription factors therefore leading to higher gene expression. Conversely histone de-acetylation by histone deacetylase (HDAC) results in reduced gene expression. Regulation of both histone acetylation and deacetylation has been associated to the action of psychoactive drugs^24^,^25^.

Here, we examined whether high-frequency rTMS applied over the frontal cortex of awaken mice can induce changes of protein expression and chromatin organization similar to those resulting from chronic drug treatment. Our results indicate that, as compared to sham, rTMS induces prolonged changes in the expression of proteins such as cyclin dependent kinase 5 (CDK5) and the post synaptic density protein 95 (PSD-95) which are both involved in modulating synaptic plasticity. These changes were accompanied by a modulation of histone acetylation at the promoter of the genes encoding these two proteins. Furthermore, these changes were dependent, on HDAC and D_2_ dopamine receptor (D_2_R) functions. These observations provide evidence for long-lasting epigenetic effects of rTMS that can be mediated, at least in part, by a modulation of dopamine neurotransmission.

## Results

### rTMS induces region specific long lasting changes of protein levels

**Figure 1:**
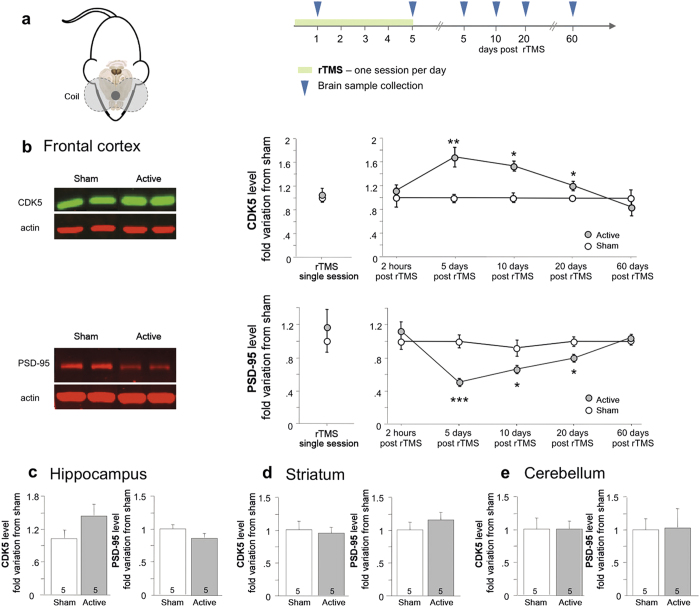
Long lasting changes induced by rTMS in the frontal cortex. (**a**) Experimental design for active rTMS protocol and brain collection. (**b**) Level expression of CDK5 and PSD-95 were quantified by western blot in the frontal cortex of stimulated or sham mice at different time point (n = 5 per group). Representative blots correspond to brain extracts removed 5 days after the last stimulation. Level expression of CDK5 and PSD-95 were quantified 5 days after the end of the rTMS protocol in the hippocampus (**c**), the striatum (**d**) and the cerebellum (**e**). Data represent mean ± S.E.M of fold change of protein expression level normalized to sham. Numbers at the bottom of the columns represent the number of mice per group. *p < 0.05, **p < 0.01 and ***p < 0.001. as compared to sham. Panel (**a**) was drawn by A. Etiévant.

**Figure 2:**
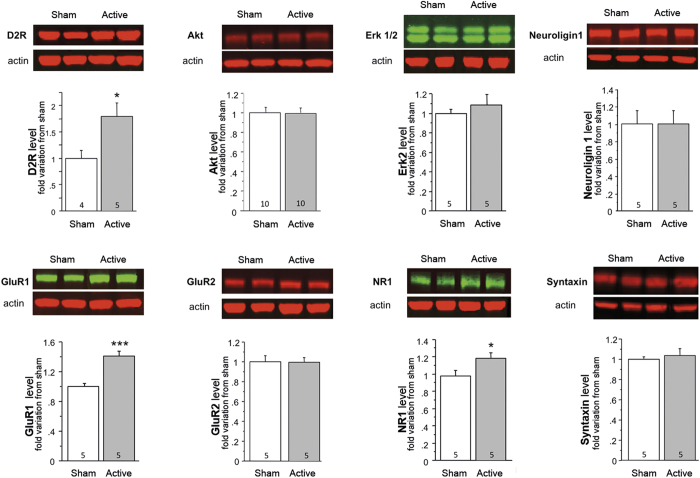
rTMS induced changes in expression level of several protein involved in synaptic plasticity. Western Blot analysis of frontal cortex extracts removed 5 days after the last stimulation from stimulated or sham mice. Data represent mean ± S.E.M of fold change of protein expression level normalized to sham. Numbers at the bottom of the columns represent the number of mice per group. *p < 0.05, **p < 0.01 and ***p < 0.001 compared to sham using unpaired student t test.

Awaken mice received active (15 Hz) or sham rTMS daily for 5 consecutive days over the frontal cortex (Fig. 1a). Western blot analyses were conducted following rTMS to examine the possible consequences on the levels of several proteins chosen on the base on their known implication in synaptic plasticity and related neuronal cell signaling^24^,^25^. These analyses revealed (Fig. 1b) that 5 sessions of active as compared to sham rTMS reduced the expression of the synaptic scaffolding protein PSD-95 (p = 0.0005) while increasing levels of the cyclin-dependent kinase 5 (CDK5, p = 0.004) 5 days after the last stimulation. These changes were not observed directly after the last rTMS session (2 h) or after a single session of rTMS (Fig. 1a). In addition, 5 sessions of rTMS caused an increase in levels of glutamate receptors subunits GluR1 (p = 0.002) and NR1 (p = 0.042) as well as the dopamine D_2_R (p = 0.041) 5 days after the rTMS session whereas levels of the other tested proteins remained unaffected (Fig. 2).

Upregulation of CDK5 and PSD-95 downregulation have both been associated with prolonged genetic or repeated pharmacological stimulation of dopamine neurotransmission^26^,^27^,^28^,^29^ and contribute to behavioral consequences of drug treatment. Interestingly, rTMS has been shown to stimulate dopamine neurotransmission within specific brain areas of human^14^,^15^,^17^, monkey^16^ and rodent^17^. Hence, we elected to further examine the effects of rTMS on CDK5 and PSD-95 protein levels. As shown in Fig. 1b, active as compared to sham rTMS induced a significant increase in CDK5 protein level in the frontal cortex 5 days (p = 0.0046), 10 days (p = 0.0014) and 20 days (p = 0.037) after the last stimulation session. In addition, active rTMS led to a significant decrease of PSD-95 levels in the frontal cortex 5 days (p = 0.0005), 10 days (p = 0.045), and 20 days (p = 0.027) after the last rTMS session as compared to sham rTMS. Levels of CDK5 and PSD-95 returned to baseline after 60 days (p = 0.44 and p = 0.35, respectively). Remarkably, these changes in CDK5 and PSD-95 protein levels were confined to the stimulated area, as levels of these proteins were not affected in the striatum (p = 0.27 and p = 0.41 respectively), hippocampus (p = 0.15 and p = 0.22 respectively) or cerebellum (p = 0.23 and p = 0.93 respectively; Fig. 1c). Furthermore, there was no significant difference in GFAP protein levels (p = 0.74) nor NeuN labeling (p = 0.47) in the frontal cortex between stimulated and sham mice (Figure S1), suggesting that rTMS did not trigger neuroinflammation nor neuronal loss within the stimulated area.

### A contribution of D_2_ dopamine receptors to the effects of rTMS on PSD-95 and CDK5 levels

**Figure 3:**
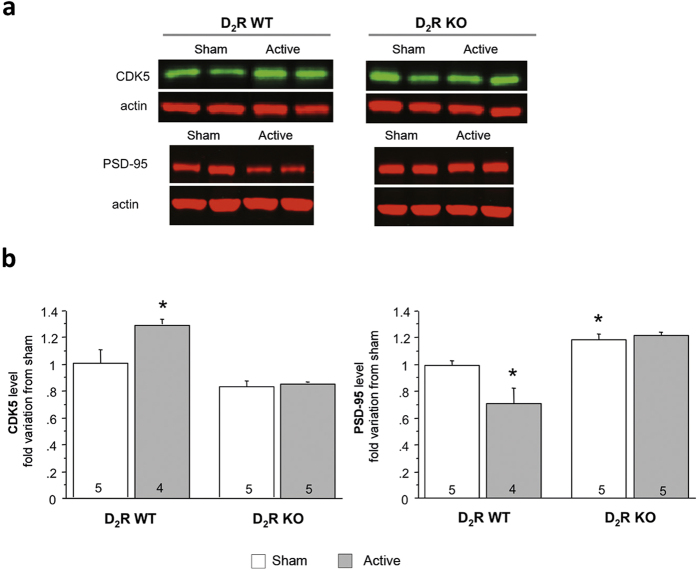
rTMS-induced changes are dependent of the dopaminergic system. **(a)** Representative Western Blot analysis performed with frontal cortex extracts of WT and D_2_R-KO animals after rTMS protocol (active, sham). (**b**) Levels of CDK5 and PSD-95 were quantified per western blot in WT or KO mice 5 days after the last stimulation session. Data represent mean ± S.E.M of fold change of protein expression level normalized to sham. Numbers at the bottom of the columns represent the number of mice per group. *p < 0.05 compared to WT sham.

Given that rTMS increased levels of dopamine D_2_R in the frontal cortex (Fig. 2) and that CDK5 and PSD-95 expression can be regulated by dopamine neurotransmission, we then examined the effects of rTMS on these two proteins by using D_2_R-KO mice (Fig. 3). For CDK5, a two-way ANOVA revealed a significant main effect of treatment (F(1,15) = 6.58; p = 0.021) and mice genotype (F(1,15) = 25,11; p = 0.0002) and a significant interaction (F(1,15) = 4.42; p = 0.046). Concerning PSD-95, a two-way ANOVA revealed a significant main effect of treatment (F(1,15) = 4.62; p = 0.048) and mice genotype (F(1,15) = 35,33; p < 0.0001) and a significant interaction (F(1,15) = 7.85; p = 0.013). As expected, levels of CDK5 and PSD-95 were respectively increased (p = 0.046) and decreased (p = 0.035) by rTMS in WT littermates of D_2_R-KO mice. However, rTMS did not induce changes of PSD-95 (p = 0.45 vs sham) and CDK5 (p = 0.60 vs sham) protein levels 5 days after the last stimulation in D_2_R-KO mice, indicating that rTMS-induced changes of CDK5 and PSD-95 protein levels were dependent on D_2_R expression.

### Epigenetic regulation of CDK5 and PSD-95 in response to rTMS

**Figure 4:**
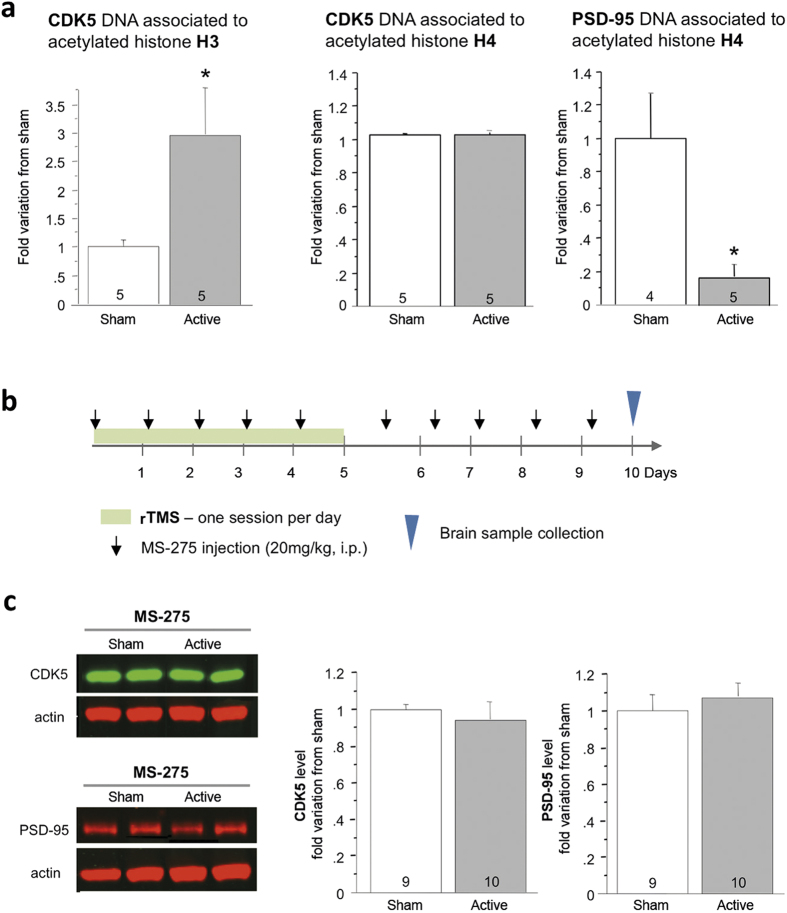
rTMS induced epigenetic changes of CDK5 and PSD-95 gene expression. (**a**) Effects of active and sham rTMS on histone H3 and H4 acetylation at the CDK5 and PSD-95 gene promoters in the frontal cortex 5 days after the last stimulation session. (**b**) Experimental design for rTMS, the administration of MS-275 and brain collection. (**c**) Level of CDK5 and PSD-95 were quantified per western blot in the frontal cortex after a chronic administration of MS-275 or vehicle. Data represent mean ± S.E.M of fold change of protein expression level normalized to sham. Numbers at the bottom of the columns represent the number of mice per group. *p < 0.05 and **p < 0.01.

Increased stimulation of D_2_R has been associated with changes of gene expression following epigenetic reorganization of chromatin structure through histone acetylation^30^,^31^. We thus explored whether rTMS-induced changes in CDK5 and PSD-95 levels may result from an epigenetic modulation of their expression associated to changes of histone acetylation. We analyzed histone acetylation in the promoter region of CDK5 and PSD-95 gene in the frontal cortex of stimulated- and sham-treated mice using a chromatin immunoprecipitation (ChIP) assay. As shown Fig. 4a, active as compared to sham rTMS significantly increased histone H3 acetylation in the promoter region of CDK5 (p = 0.043), which is in agreement with an enhanced CDK5 expression in the frontal cortex. The amount of PSD-95 promoter DNA associated with acetylated histone H3 was too low to be quantified. Conversely, whereas there was no change of histone H4 acetylation at the *cdk5* gene promoter compared to sham (p = 0.83), active rTMS induced a decreased acetylation of histone H4 at the PSD-95 promoter (p = 0.012), which is compatible with decreased expression of the PSD-95 gene. To further establish the contribution of epigenetic mechanisms, the class 1 HDAC inhibitor MS-275 (20 mg/kg i.p.) was administered before each stimulation session and daily for 5 days after the end of rTMS protocol (Fig. 4b). Remarkably, MS-275 prevented the effects of rTMS on CDK5 (p = 0.57) and PSD-95 (p = 0.54) levels in the frontal cortex of stimulated animals (Fig. 4c), while treatment with the HDAC inhibitor in the context of a sham stimulation protocol did not affect levels of these two proteins (Figure S2a).

Administration of MS-275 prevented the increase of CDK5 levels in response to rTMS. This is paradoxical since HDAC inhibitors should increase histone acetylation and gene expression. We thus conducted a ChIP assay to evaluate the impact of MS-275 on the increased of histone H3 acetylation at the *cdk5* gene promoter (Figure S2b). Repeated administration of MS-275 along with the sham rTMS protocol had no significant effect on the abundance of acetylated histone H3 at the *cdk5* gene promoter. As shown previously, active stimulation increased acetylation of histone H3 at this promoter (p = 0.008) while co-administration of MS-275 with active rTMS prevented this effect.

## Discussion

Results presented here provide *in vivo* evidence that rTMS can induce in long lasting changes of gene expression that can result from a modulation of histone acetylation taking place after several sessions of rTMS. Absence of an effect of rTMS on CDK5 and PSD-95 levels in D_2_R-KO mice further suggests that some of these changes could be mediated by an activation of dopamine neurotransmission in response to rTMS.

Epigenetic modifications are believed to play a central role in the etiology of several psychiatric illnesses, including depression, post-traumatic stress disorder and substance use disorders^32^,^33^,^34^. Conversely some psychoactive pharmacological agents and drugs of abuse have been shown to modulate the expression of myriads of genes by affecting histone acetylation. We have focused our study on two proteins encoded by such genes, PSD-95 and CDK5. This choice was inspired by the known action of repetitive stimulation of dopamine neurotransmission on the expression of these genes and on the known contribution of the proteins they encode to the regulation of neuronal functions in response to drug administration^26^,^35^. Measurement of the effect of rTMS on the expression of these two proteins and on histone acetylation at their respective gene promoters, therefore represents a good case example to establish proof of concept evidence that rTMS can regulate histone acetylation.

Stimulation of dopamine receptors has been shown to regulate the expression of several genes via epigenetic mechanisms both in striatal and frontal cortex neurons^33^,^36^. Among these genes, *cdk5* encodes for a serine threonine kinase that is upregulated in response to diverse forms of repeated treatment including electroconvulsive shock therapy^37^ and chronic psychostimulant administration^26^. Upregulation of *cdk5* gene expression as a result of chromatin reorganization following repeated dopamine receptor stimulation has also been shown to play a central role in mediating cocaine induced plasticity in the striatum^20^. The *dgl4* gene encodes for PSD-95, a synaptic molecular scaffold that is involved in the regulation of ionotropic glutamate receptor complexes in post-synaptic densities^38^. PSD-95 is downregulated in striatal medium spiny neurons in response to chronic cocaine administration as well as in genetic animal models of dopamine super-sensitivity^35^. Downregulation of PSD-95 has also been shown to contribute to an enhanced neuronal excitability in these different animal models.

The complete characterization of the mechanisms by which rTMS regulates histone acetylation at the *cdk5* and *dgl4* gene promoters will require further investigation. Results obtained for PSD-95 suggests that rTMS may induce some HDAC activity leading to reduce PSD-95 expression as a result of a deacetylation of histone H4 at the promoter of this gene. Administration of the HDAC inhibitor MS-275 would antagonize this effect of rTMS while not affecting PSD-95 expression when given with sham rTMS. Expression of CDK5 is known to be upregulated following accumulation of the transcription factor ΔFosB following chronic cocaine administration^26^. Accumulation of ΔFosB leads to a acetylation of histone H3 at the *cdk5* gene promoter^39^. It is possible that repeated rTMS sessions may engage this same mechanism. However, the observation that MS-275 prevents the regulation of CDK5 by rTMS also points toward the contribution of additional mechanisms. Indeed, MS-275 should promote histone acetylation and promote gene expression. One possibility to explain this apparent paradox is that MS-275 may have activated a negative regulator acting upstream of the *cdk5* gene promoter in the signaling cascade leading to the activation of CDK5 expression by rTMS.

Our observation of a D_2_R and HDAC activity dependent regulation of CDK5 and PSD-95 expression in the in mice stimulated with active rTMS over the frontal cortex is compatible with what is known about the regulation of these two genes by dopamine in other experimental systems. Considering the role of such variations in cocaine-induced plasticity, it is possible that CDK5 and PSD-95 may also contribute to the regulation of neuronal functions by rTMS. Moreover, our results indicate that the abundance of other proteins, including D_2_R and the ionotropic glutamate receptors subunits GluR1 and NR1, are also affected by rTMS, either directly or as a consequence of the modulation of other gene products. Further studies involving genome wide characterization of epigenetic signatures of different rTMS protocols combined with the use of several mouse models lacking targeted genes will thus be necessary to fully understand the contribution of changes in histone acetylation to behavioral outcomes and therapeutic effects. Furthermore, in addition to dopamine, some rTMS protocols have also been shown to increase BDNF release^11^,^12^,^13^, which may constitute an additional mechanism contributing to rTMS induced changes in histone acetylation.

In conclusion, regulation of histone acetylation by rTMS constitutes a potential mechanism through which noninvasive brain stimulation can result in long-term modulation of mood and behavior. It is noteworthy that repeated cocaine administration also results in prolonged reduction of PSD-95 and increased CDK5 expression^26^,^35^. Therefore, rTMS engages epigenetic mechanisms that modulate gene expression and potentially synaptic plasticity in a manner that is similar to various psychoactive drugs, irrespective of their potential for therapy or abuse. This provides a potential mechanism supporting a biological basis for the effects of rTMS. It also indicates that, like several other protracted psychoactive treatments, repeated sessions of rTMS may not be innocuous since they can exert long lasting effects on their neuronal substrates. This should certainly be taken into consideration when using rTMS in medical and non-medical settings. Understanding the regulation of chromatin organization by rTMS may result in the development of better guidelines for the safe and ethical application of rTMS as well as its combination with psychopharmacological treatments.

## Material and Methods

### Animals

Male and female C57BL/6JWT mice (Jackson Laboratory, Bar Harbor, ME) were housed by gender under a 12 h light/dark cycle with food and water *ad libitum*. Mice were aged between two and four months during experiments. To determine the involvement of dopamine neurotransmission on the effects of rTMS, the C57BL/6J D_2_R KO and WT mice (Jackson Laboratory) were used and housed in the same conditions. All procedures were conducted in accordance with the Canadian Council on Animal Care and approved by the Laval University animal care committee.

### rTMS

Both active and sham rTMS were administered daily (in the morning) for 5 consecutive days using a small figure-of-eight coil (inner diameter 2.5 cm; outer diameter 5 cm; Magstim Company Limited, UK). Stimulation consisted in 3 trains of 150 pulses delivered at 15 Hz (10 s each train) with an intertrain interval of 0.5 s, which led to a total of 450 pulses administrated during 31 s per day. The intensity was set at 53% of the maximal output of a Magstim Rapid2 stimulator. For the active condition, the coil was placed flat on the scalp over the frontal cortex of hand-restrained mouse. For the sham condition, the coil was placed with a perpendicular angle on the scalp over the frontal cortex of hand-restrained mouse. Mice were acclimatized to hand restriction to minimize stress. Each mouse was manually handled 5 times during 40 s for 4 days before the first rTMS session. rTMS did not induce seizure, sign of discomfort or any apparent behavioral changes. To determine the acute effects of rTMS, mice’s brains were collected immediately after a single rTMS session. To evaluate long lasting effects of rTMS, brains were collected 2 hours and 5, 10, 20 or 60 days after the last stimulation session.

### Drugs administration

Administration of the HDAC inhibitor MS-275 (Selleck Chemicals, Houston, TX) was done as described^40^. This drug regimen was chosen on the base of its low toxicity and compatibility with systemic administration. Briefly, the drug was dissolved in a solution of saline 0.9% containing 3.5% of DMSO and injected (i.p.) at a dose of 20 mg/kg per day. MS-275 or vehicle was administrated during 10 days: one injection 1 h30 before the stimulation during the 5 days of the rTMS protocol (active, sham) and one injection every morning for 5 days after the last stimulation session.

### Western Blot analyses

Brains of stimulated and sham mice were collected as previously described^29^. In each mouse, the frontal cortex, striatum, hippocampus and cerebellum were dissected, and frozen in liquid nitrogen before protein extraction. Tissue samples were homogenized in boiling 1% sodium dodecyl sulfate (SDS) and boiled for 5 min. Protein concentration was measured using a DC-protein assay (Bio-Rad, Hercules, CA, USA). Protein extracts (25 μg) were separated on 10% SDS/PAGE Tris-glycine gels (Life-Technologies, Burlington, Ontario) and transferred to nitrocellulose membranes (Life-Technologies). Blots were immunostained overnight at 4 °C with primary antibodies. Immune complexes were revealed using appropriate IR-dye-labelled secondary antibodies. Quantitative analyses of fluorescent IR dye signal were carried out using an Odyssey Imager (Licor biotechnology, Lincoln, NE). For quantification, actin was used as a loading control for the evaluation of total protein levels. Results were further normalized to respective control conditions.

### Immunohistochemistry

Mice were deeply anaesthetised with ketamine-xylazine and transcardially perfused with 4% paraformaldehyde in phosphate-buffered saline (PBS). Post-fixed brains were sectioned (40 μm sections, coronal and sagittal sections) and stored in PBS containing 0.1% of sodium azide at 4 °C. Free-floating sections were then incubated for 2 h in blocking buffer (1% BSA in 0.5% Triton X-100, PBS) and exposed overnight (4 °C) to the primary antibodies. After several washes, sections were exposed during 2 h to the IR Dye 680 labeled secondary antibodies at room temperature. After washing, slices were mounted with Prolong gold antifade reagent (Life-Technologies). Quality of staining was verify using a Zeiss Axioimager M1M fluorescence microscope equipped for signal detection of fluorescence emission at 700 nm. Sections were then scanned at a resolution of 20 μm using the Odyssey imager and quantification of IR Dye 680 fluorescence signal was performed in the stimulated region as previously described^41^.

### Antibodies

For Western blot analyses, the following primary antibodies were used: mouse anti-PSD-95 (1:250; BD transduction), rabbit anti-CDK5 (1:500; Santa Cruz biotechnology, Dallas TX), mouse anti-Akt (1:1000, Cell signal technology, Danvers, MA), rabbit anti-p44/42 MAPK (Erk1/2) (1:1000; Cell signal technology), mouse anti-GFAP (1:500, Sigma Aldrich, Oakville, Ontario), mouse anti-syntaxin (1:10 000, Sigma Aldrich), rabbit anti-GluR1 (1:2000, Millipore, Billrica, MA), mouse anti-GluR2 (1:2000, Millipore), mouse anti-NR1 (1:2000), rabbit anti-D2R (1:500, Millipore) and mouse anti-actin (1:10 000; Millipore). Secondary antibodies IRDye 680 Goat Anti-Rabbit IgG (1:10 000; Mandel Scientific, Guelf, Ontario) or IRDye 800 Goat Anti-Mouse (1:10 000; Mandel Scientific) were then used.

For immunochemistry analysis, mouse monoclonal anti-neuronal nuclei (NeuN) (1:250; Millipore) and mouse monoclonal anti-actin (1:5000; Millipore) were used as primary antibodies. Revelation of labeling using the Odyssey imager was performed using IRDye 800 Goat Anti-mouse IgG (1:1000) and IRDye 680 Goat Anti-mouse IgG (1:1000) secondary antibodies.

### Chromatin Immunoprecipitation

ChIP assays for acetylated histone H3 and acetylated histone H4 were performed according to a protocol from Epigentek (Farmingdale, NY). Briefly, chromatin extracted from the frontal and prefrontal cortex of mice was sheared by sonication, and then immunoprecipitated using anti-acetylated H3 or anti-acetylated H4 antibodies in micro-wells. Captured DNA is then released from the antibody/acetylated histone complex, reversed and purified. Levels of specific histone modifications at each gene promoter of interest (CDK5 and PSD-95) were determined by measuring the amount of that gene in chromatin immunoprecipitates by quantitative PCR using a Lightcycler^TM^ (Roche Life Science, Laval, Quebec). The primers used were as follows: For CDK5 GCGTTGCAGAGGAGGTGGTA and CGCAGCCTGTTGGACTTTGT, for PSD-95 GAGGGGAAGGAGAAGGTTG and CCCCTACCCCTCCTGAGAAT. The primers used for β-actin control promoter were CTCTCAGCTGTGGTGGTGAA and AGCCATGTACGTAGCCATCC. Input DNA (non immunoprecipitated) and immunoprecipitated DNA were amplified by PCR in the presence of Taq polymerase (Sigma Aldrich). Each PCR reaction was run in triplicate for each brain sample.

### Statistical analysis

All results were expressed as means ± standard errors of the mean (S.E.M). Unpaired Student’s *t*-test was used to evaluate the effects of rTMS at different time points on protein level expression in the frontal cortex, the hippocampus, the striatum and the cerebellum. Unpaired Student’s *t*-test was also used to evaluate the effects of rTMS on histone H3 and H4 acetylation in the promoter region of CDK5 and PSD-95 and the consequences of MS-275 injection on rTMS-induced change in protein expression. Two-way ANOVA was used to analyze the effects of rTMS on D_2_R-KO mice. Any overall statistical differences were further analyzed using Fisher’s post hoc test. Statistical differences were set at p < 0.05, using Prism 4.0 (GraphPad software inc, San Diego, CA).

## Additional Information

**How to cite this article**: Etiévant, A. *et al.* Repetitive transcranial magnetic stimulation induces long-lasting changes in protein expression and histone acetylation. *Sci. Rep.* **5**, 16873; doi: 10.1038/srep16873 (2015).

## Supplementary Material

Supplementary Information
