# Supplementary material for: Repetitive transcranial magnetic stimulation induces long-lasting changes in protein expression and histone acetylation
Source: Sci Rep. 2015 Nov 20;5:16873. doi: 10.1038/srep16873 (PMC4653621; doi:10.1038/srep16873)

Repetitive transcranial magnetic stimulation induces long-lasting changes in protein expression and histone acetylation

Adeline Etiévant[1], Stella Manta[1], Camille Latapy[1], Luiz Alexandre V. Magno[1,2], Shirley Fecteau[1,3*] and Jean-Martin Beaulieu[1*]

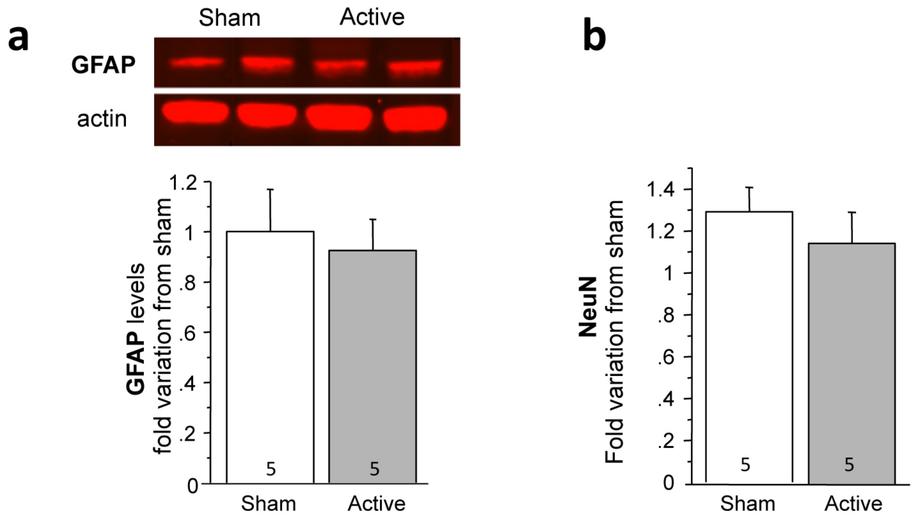

Figure S1 rTMS does not result in structural brain alterations in mice.

(a) Levels of GFAP were quantified using Western Blot in the frontal cortex of stimulated or sham mice. There was no inflammatory reaction in the stimulated area. (**b**) NeuN labeling using immunohistochemistry followed by signal fluorescent quantification revealed no neuronal loss in stimulated frontal cortex. Data represent mean ±S.E.M of fold change of protein expression level normalized to sham Numbers at the bottom of the columns represent the number of mice per group.

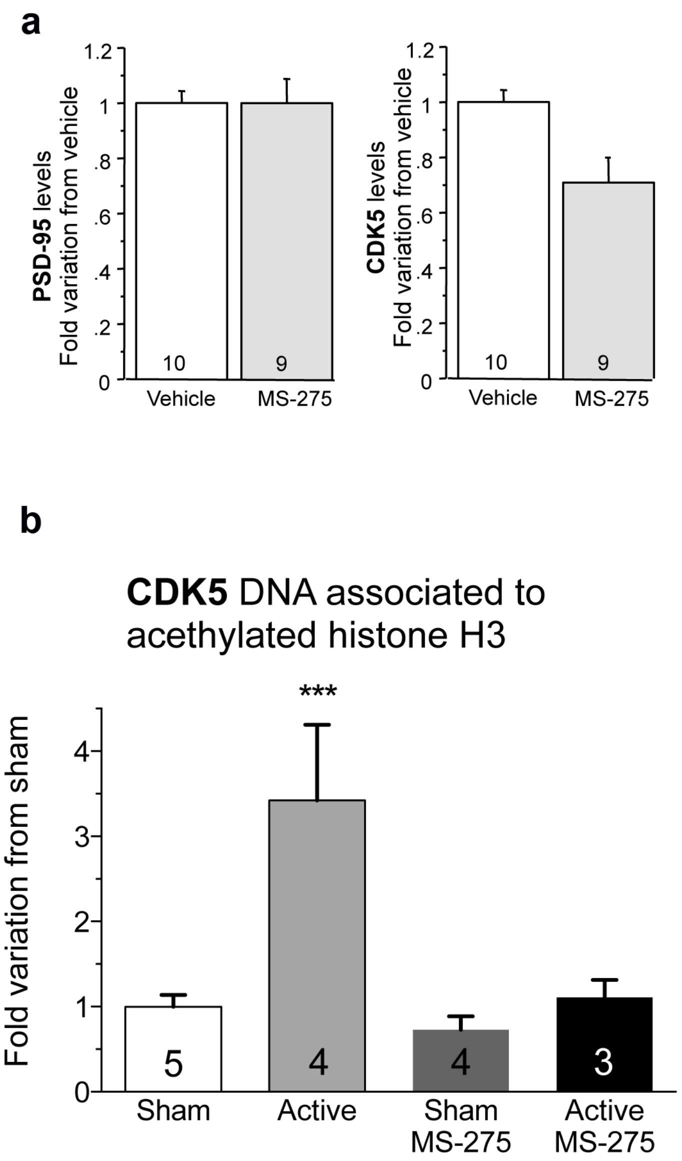

Figure S2. Control parameters for epigenetic study.

(**a**) Effect of MS-275 administration on levels of CDK5 and PSD95 were quantified per western blot 5 days after the last sham stimulation session. Data represent mean ±S.E.M of fold change of protein expression level normalized to vehicle. Numbers at the bottom of the columns represent the number of mice per group. No significant differences were found between groups, student T test.

(b) Effects of active and sham rTMS with or without administration of MS-275 on histone H3 acetylation at the CDK5 gene promoters in the frontal cortex 5 days after the last stimulation session. Data represent mean ±S.E.M of fold change normalized to sham. Numbers at the bottom of the columns represent the number of mice per group. ***$p<0.005$, one-way anova as compared to sham.

UNEDITED GELS OF WESTERN BLOT USED IN THE PUBLICATION

Figure 1. Long lasting changes induced by rTMS in the frontal cortex.

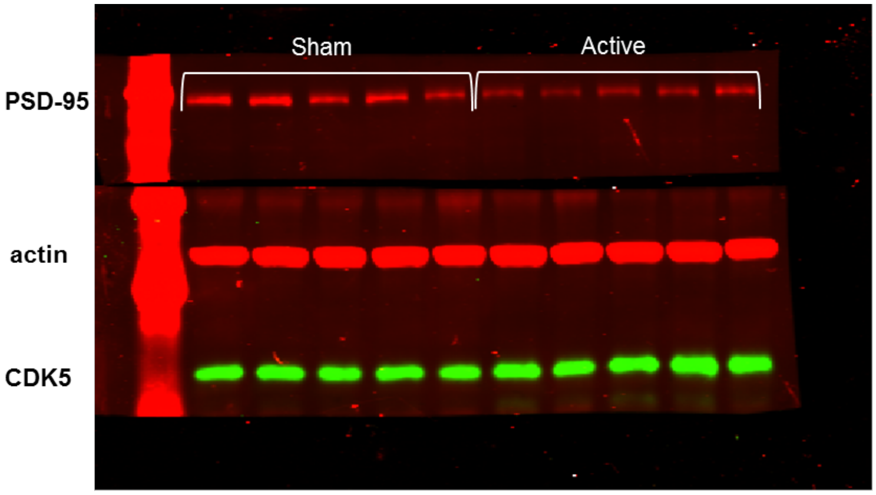

Figure 2. rTMS induced changes in expression level of several protein involved in synaptic plasticity.

For GluR1:

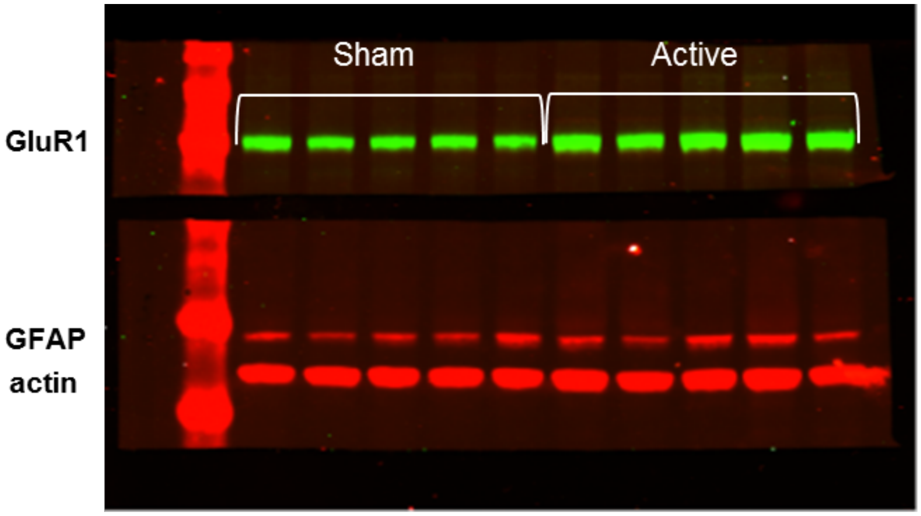

For NR1, GluR2, D2R and Erk 1/2:

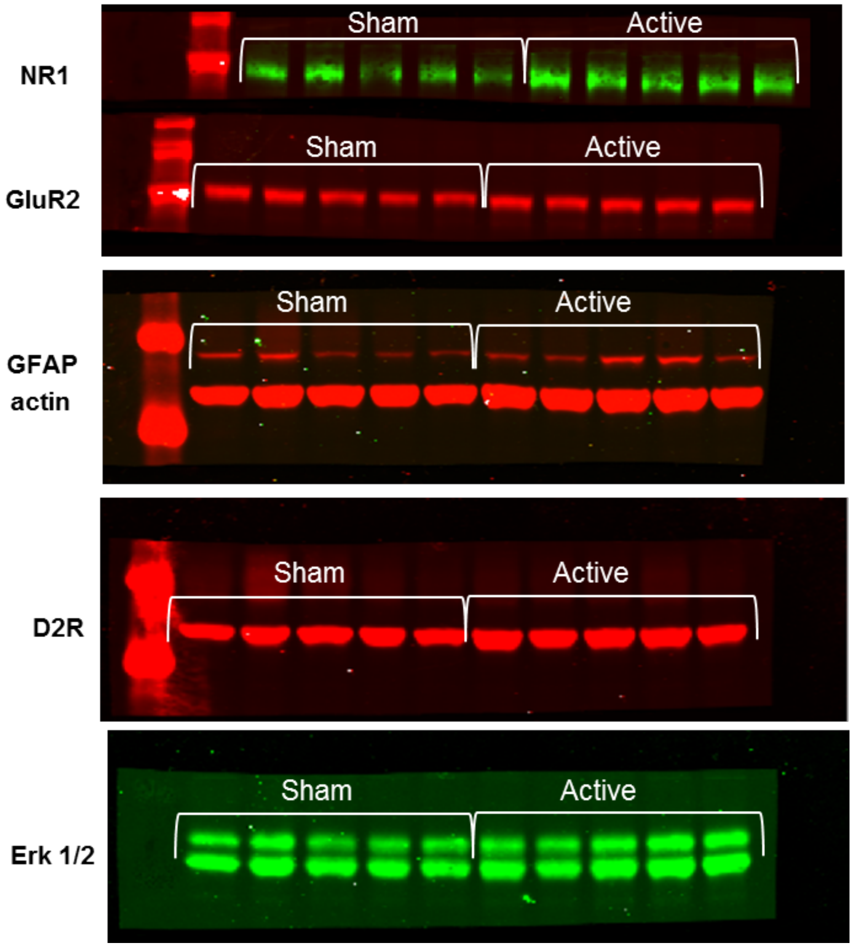

For Neuroligin1 and Akt:

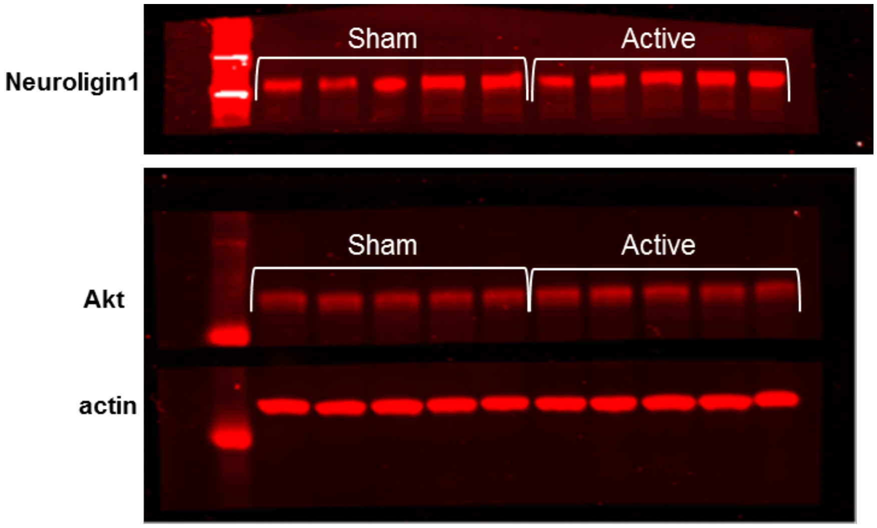

For syntaxin:

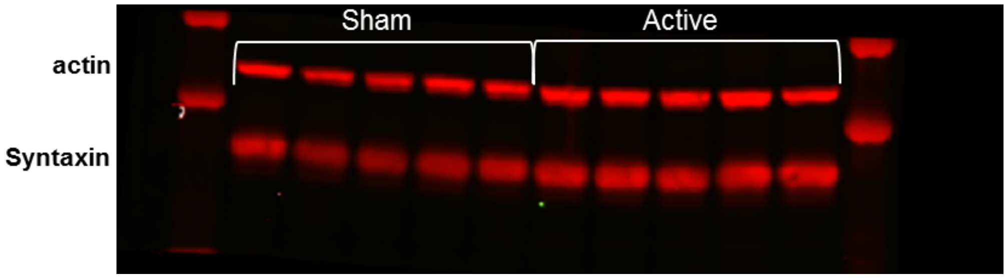

Figure 3. rTMS-induced changes are dependent of the dopaminergic system.

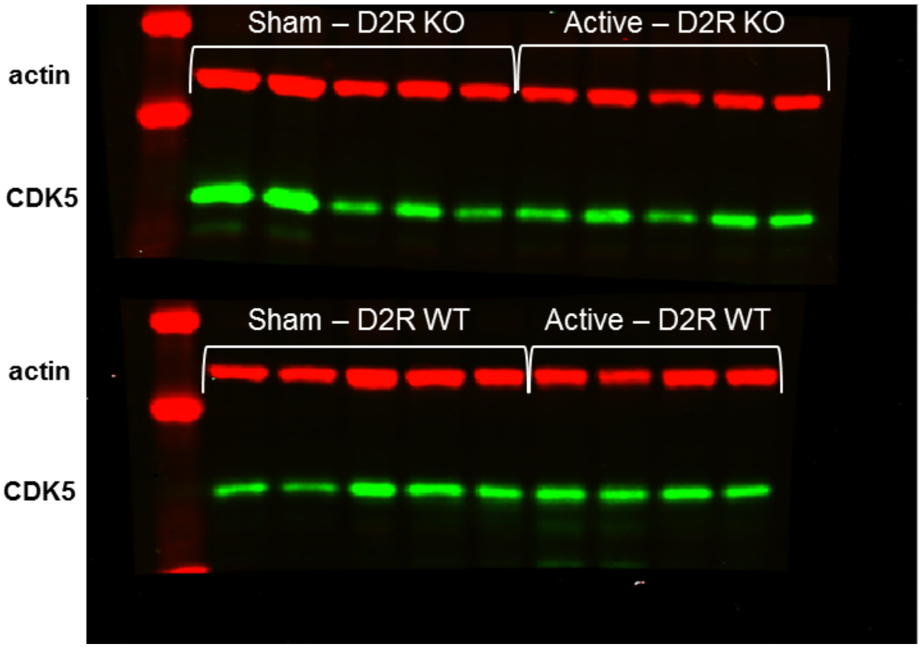
Sham – D2R KO
Active – D2R KO
actin
CDK5
Sham – D2R WT
Active – D2R WT
actin
CDK5

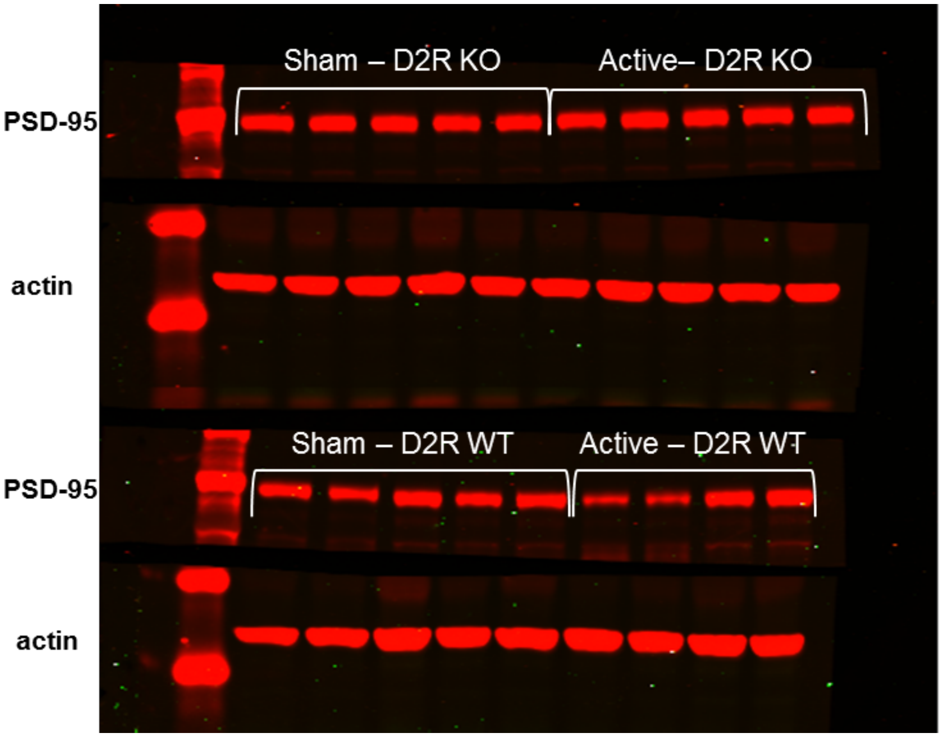
Sham – D2R KO
Active– D2R KO
PSD-95
actin
Sham – D2R WT
Active – D2R WT
PSD-95
actin

Figure 4. rTMS induced epigenetic changes of CDK5 and PSD95 gene expression.

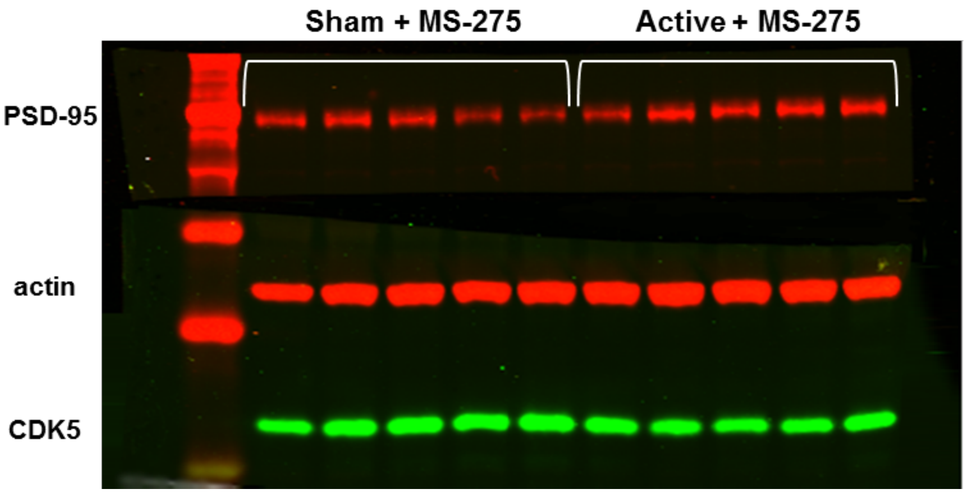

Supplement: Supplementary Information [file srep16873-s1.pdf]
